# Managing community engagement in research in Uganda: insights from practices in HIV/AIDS research

**DOI:** 10.1186/s12910-022-00797-6

**Published:** 2022-06-14

**Authors:** John Barugahare, Nancy E. Kass

**Affiliations:** 1 Department of Philosophy, Makerere University, Kampala, P. O. Box 7062, Kampala, Uganda; 2 Johns Hopkins Berman Institute of Bioethics and Johns Hopkins Bloomberg School of Public Health, 1809 Ashland Avenue, Baltimore, MD 21205 USA

**Keywords:** Community engagement, Research ethics, Community advisory boards, Methods, Strategies, Approaches

## Abstract

**Background:**

Community engagement (CE) in research is valuable for instrumental and intrinsic reasons. Despite existing guidance on how to ensure meaningful CE, much of what it takes to achieve this goal differs across settings. Considering the emerging trend towards mandating CE in many research studies, this study aimed at documenting how CE is conceptualized and implemented, and then providing context-specific guidance on how researchers and research regulators in Uganda could think about and manage CE in research.

**Methods:**

We conducted qualitative interviews and focus group discussions involving forty-one respondents who were experienced in HIV/AIDS biomedical research involving CE. Thirty-eight of these were directly or indirectly associated with Uganda’s leading research institution in the field of HIV/AIDS. They included Principal Investigators, Community Liaisons Officers, Research Ethics Committee members and Community Advisory Board Members. Three respondents were from Uganda National Council for Science and Technology. Data were collected between August 2019 and August 2020, using audio-taped focus group discussions and key informant interviews, transcribed and analyzed manually to generate themes and subthemes.

**Results:**

Three major themes emerged: goals or value of CE; the means of CE, and, the evaluation of CE. Goals or value of CE generated four subthemes representing the overarching goals of CE: (1) Promote communities’ agency; (2**)** Generate and sustain trust; (3) Protect and promote communities’ rights and interests; and, (4) Help studies optimize participation in the form of enrolment and retention of participants. What usually comes under the nomenclatures of methods, strategies, and approaches of CE, such as town-hall meetings, sports events, drama, and the like, should simply be understood as the means of CE, and it is not desirable to hold pre-conceived and fixed ideas about the best means to conduct CE in research since a lot depend on the context. Finally, the study found that despite CE’s critical importance, which suggests the need to track and evaluate it, CE is currently intermittently evaluated, and for inadequate motivations.

**Conclusions:**

Existing guidance on how to conduct robust CE in research is no substitute for creativity, flexibility, and reflexivity on the part of both researchers and research regulators.

**Supplementary Information:**

The online version contains supplementary material available at 10.1186/s12910-022-00797-6.

## Background

There is wide consensus on the value of community engagement (CE) in research [1–10]. According to these views, CE is valuable for both intrinsic and instrumental reasons – ensuring respect for communities’ ethical and cultural norms and values; ensuring that research addresses the needs and priorities of communities; ensuring ease of recruitment and retention of study participants; and increasing chances of uptake of research results by the concerned communities, among others. In Uganda, while the idea of CE in research dates at least as far back as the early 1990s [11], it is in about the last decade that this idea has gained more traction, mostly in HIV/AIDS research [12–16]. Currently the Uganda National Council for Science and Technology (UNCST) is finalizing guidelines that will make CE a requirement in all research involving human participants where procedures and results could affect communities’ interests and the environment. These guidelines require researchers to develop CE plans which will be subject to review by Research Ethics Committees (RECs) as part of research protocol review. Further, researchers will be required to monitor and evaluate the success and methods of their CE processes. In order to facilitate rigorous processes of CE in research, this study aimed at analyzing existing knowledge and experiences in CE in Uganda to provide locally nuanced insights and develop context-specific guidance for planning, reviewing and evaluating CE in research in Uganda. In this paper we report views and insights about how and why CE is implemented in HIV/AIDS biomedical research in Uganda, as well as infer from these practices what may work best in CE in research generally.

Generally, both at international and national levels, there has been strong encouragement for CE in community-based research (and arguably all research that has potential to affect communities’ interests), and guidance has been provided to that effect [17–22]. Recently, however, CE has been indicated as necessary for all health research [23, 24], with a number of suggestions on how this process can be undertaken [25–33]. Yet in addition to local and international guidance and lessons from experiences in other contexts [30, 34–40], to be locally sensitive in Uganda, such guidance would need to be partly based on a wealth of experience gained over many years of CE practice in research in that location. This study sought the experiences and views related to CE of Principal Investigators (PIs), Community Advisory Board (CAB) members in HIV/AIDS biomedical research, and Research Ethics Committee (REC) members of the leading HIV research institution in Uganda – the Uganda Virus Research Institute (UVRI).

## Methods

### Study design

This was a qualitative case study of CE practices in HIV/AIDS research at the UVRI and closely affiliated research institutions and individual projects in Uganda. This design was deemed the most appropriate because this study aimed at gaining a deeper understanding of concrete practices in CE in HIV research and how those insights could inform CE in research generally in the specific context of Uganda. The case study, the UVRI, is a leading research institution in HIV biomedical research in Uganda.

### Study population

The study population included those involved in HIV/AIDS biomedical studies in Uganda in which CE was conducted. Specifically participants included CAB members of HIV/AIDS research institutions and individual research projects; Community Liaisons Officers (CLOs); PIs for HIV/AIDS biomedical research projects at UVRI; UVRI Research Ethics Committee (REC) members and some officials at the country’s research regulatory authority – UNCST.

### Data collecting tools

Three data collecting tools were developed: a Focus Group Discussion (FGD) guide to explore the experiences and perspectives of CAB members on goals, experiences and best practices in CE; a Key Informant Interview (KII) guide to collect views from institutions’ and individual projects’ CLOs and PIs in HIV/AIDS research; and another FGD guide in the form of a hypothetical matrix for guiding the planning, reviewing and evaluating CE. A draft matrix was developed as a synthesis from the literature of the various scholarly views, experiences, and official guidance on good practices in CE (we will report on the matrix separately). The major themes in all data collection related to general experiences in CE; the goals of community engagement; categories of community stakeholders that should be included and why; approaches/strategies/methods of CE, the processes involved and activities involved; challenges encountered in CE; and strategies for minimizing and resolving those challenges. Some of the participants were given the draft matrix to study and comment on its clarity, and were asked about its comprehensiveness and simplicity based on perceived ease of use. We proposed the draft CE matrix to these respondents as a summary of how to systematically think about CE, with hope that the matrix would help those who review it to quickly detect and fill gaps in the process of what might be described as robust CE in research.

## Sample size and sampling procedure

The study involved forty-one (41) participants. The sample size was determined by the principle of data saturation; that is, a point at which no new ideas were being generated from additional interviews. Participants were purposively selected on the basis of their participation in CE in HIV/AIDS biomedical research, and knowledge of research processes. REC members at UVRI, officials at UNCST, CLOs, and PIs were identified and approached through their institutions and requested to participate in the study. CAB members were identified and approached through the PIs and, or CLOs. The study conducted five (5) FGDs. Three of these were with CAB members. Two focus groups had eight (8) participants each, while the third had nine (9). The fourth FGD was conducted with three (3) participants from UNCST. Each of these three participants reviewed the draft matrix independently, before the FGD, and then met with others in an FGD to compare notes and work out a consensus. The fifth FGD was conducted with eight (8) members of the UVRI REC, also to review the hypothetical matrix. The study further conducted three (3) KIIs with PIs of HIV/AIDS biomedical research involving CE, and two (2) KIIs with CLOs, one from UVRI and another from a research project affiliated to UVRI. The age range for the study participants was 25 to 60 year of age, while gender representation was 23 and 18 for females and males respectively.

### Data collection and analysis

Data were collected between August 2019 and August 2020. Interviews and focus groups were audio recorded and notes were taken. For KIIs, average time was about 1 hour, while for FGD, time was about 1 hour and 30 minutes. All data were collected in English language. Audio recordings were later transcribed. Data from FGDs with CAB members and KIIs was manually analyzed to develop themes and subthemes. Data was coded using the various sections of the data collecting tools, which were later used to inductively generate major themes. Upon reading and re-reading the data transcripts, some of the major themes were collapsed into each other and re-named. This was followed by the selection of the most representative quotes for each subtheme. The results from KIIs and FGDs with CAB members whose data collecting tools did not contain the draft matrix for planning and reviewing CE, were read along those from FGDs with REC members and UNCST officials who reviewed and commented on the draft matrix to see whether perspectives differed from each other.

### Quality control

The data collecting tools were developed by the authors of this paper on the basis of views from literature. The tools were subjected to review at two separate post-doctoral research seminars at the second author’s institution of affiliation, and later subjected to pre-test among faculty conversant with CE practices at the first author’s institution. The research ethics committee also made some comments on the tools, although the comments did not suggest any radical changes. Generally this processes helped introduce some new themes in the tools and refining the phrasing of the tools.

## Study findings

From analysis of our data, there emerged three (3) major themes: (1) the goals and, or value or importance of CE; (2) the means of CE, as a generic description of strategies, approaches, activities and methods of CE; and, (3) the evaluation of the success of CE. These themes and data in each of them had similarities and differences with those we identified from literature. Each of these themes will be discussed in greater detail below.

### Goals and, or Value of CE

On the goals and value of CE, one of the major findings of this study was that there is noteworthy overlap between goals of CE as stated in the literature, and the perspectives of participants in this study. However, there was disagreement among our respondents on whether there should be some universal goals of CE, and if so, what these should be. Another major observation from the findings of this study is that in thinking about CE there is substantial overlap between the goals of CE on the one hand, and the value or importance of CE on the other. The findings from this study suggest that these are different ways of saying the same thing. A point of universal agreement from respondents was on the critical importance of communities’ *agency*; that is, that community engagement should aim at amplifying a community’s voice and influence in research by giving communities an opportunity to participate in the research process, especially the design of and translation of informed consent, community mobilizations, community education and sensitization among others.

For clarity, however, it is important to note that the views which emerged under this theme indicate that most of the goals and, or value of CE can be subsumed under four (4) sub-themes: (1) To respect and promote communities’ agency; (2) To increase participants’ and communities’ trust in the study being conducted and future studies; (3) To protect and promote individual study participants’ and their communities’ rights, interests and general well-being; and, (4) To help the study achieve its own goals.

#### Agency-promoting goals

The idea of CE as a means to respect and promote communities’ agency was widely expressed in the form of communities’ desire to participate in and influence efforts to solving their own problems*.* Some CAB members felt that as members of the community they deserve an opportunity to participate in the search for solutions to problems that afflict them, in this case the HIV/AIDS scourge. Hence, their membership to a CAB and generally participation in CE activities were described as an opportunity to contribute to efforts to solve their community’s problems*.* One of the CAB members stated:

“[…] so that’s why some of us entered to fight against AIDS and to help our people in the area.” FG01-02.

In particular, all CABs indicated that they facilitate the design of appropriate study materials, and they usually advise researchers on the appropriateness of such materials as consent form, data collecting tools, design of information materials for community education and mobilization such as posters, among others. This view was corroborated by some of the PIs.

Some of our respondents alluded to the value of agency when they described CE as a strategy for community acceptance and ownership of research. Some suggested that the goal of some CE activities is to ensure that communities develop a sense of ownership and support for the research project. In their view, it is this active involvement of communities and acceptance of their influence that create a sense of ownership of the studies.

“We take the whole day in a specific place and talk to people; some activities are carried out so that these people can own because they need to own the system otherwise people will never work with you if they don’t own what you are doing […].” FG03-04.

#### Trust-building goals

Almost all of our respondents’ views suggested that whether researchers will be trusted by the research communities partly depends on how and through whom they approach the target community. Several respondents described a key goal of CE as providing a link between communities and researchers or research institutions, a link that generally does not exist. Hence, some respondents explicitly stated one of the goals of CABs as being to bridge the gap between communities and researchers, as a strategy for improving communication between the two groups. This view was reiterated by one of the PIs, according to whom, CE facilitates community entry and hence, some of the CE activities and strategies should be driven by this goal. This PI indicated that one of the aims of CE is to create a trustful and supportive relationship with community leaders as the community gate keepers, emphasizing that such a relationship is very key especially when one goes “to the community for meeting or there is a problem or whatever it is very easy to contact these people who will help you and advise you on how to go about with whatever activity you are planning […]” (PI – 02).

One of the major and, arguably, the most critical trust-affecting feature in the researcher-community interaction are perceptions –including any myths and misconceptions-- individuals and communities have about the goals of the study. Some CAB members believed that identifying and dispelling myths was one of their best achieved goals in CE. We heard that:

“[…] You know it’s not easy because sometimes people are very scared. They hear some things from some people which make them suspicious and they say let others join; why me? So I think another achievement we can talk about in this board is clearing misconceptions and myths.” FG02-06.

One of the PIs emphasized this view with the following response:

“[…] For instance, there are myths going on in communities around that we are infecting people with HIV so am informed so we sit down as a team and we see how to go about sorting this with this problem.” (PI-02).

#### Protection of individuals and communities

Another set of goals and, or value of CE in research emerged as ensuring that the rights, interests, and well-being of individual study participants as well as those of study communities are respected, protected and promoted. Some respondents indicated that they did not want researchers to abuse the rights of their people, especially due to people’s vulnerability created by ignorance. Another threat from which the communities need to be protected, in the view of our respondents, is the potential violation of their societal norms by the researchers.

“Basically, we always tell them to first of all respect human participants, it’s very important never bring a research that is harmful to the human subjects. Secondly, they should ever respect the community norms so don’t bring something that will bring the touch the norms […].” (CLO-02)

#### Participation-optimizing goals

A substantial number of the goals of CE cited by the study participants indicated that most of the CE activities and the manner of their implementation are aimed at helping the study achieve its target on enrolment and retention of study participants. This was corroborated by a universal agreement among our respondents that the linking or bridging aim is important to ensure effective mobilization of communities to accept and participate in the study. This view was shared by CAB members, PIs as well as CLOs. In the words of some of the CAB members:

“Then secondly we help our community because we know if we are not involved as leaders these people may find it difficult to get participants. So we have mobilized our members and encourage them to support the project and enter the study hence we can find solutions to our problems and the whole country at large.” FG03-04.

This goal was emphasized by some of the PIs in the following words:

“[…] without a community engagement plan I don’t know if you are able to have conducted clinical trials because it starts with them from the communities so by the time they come here to the site to be screened and enrolled, there is a lot that has happened in the community” (PI-01).

To one of the CLOs, the goal of ensuring the successful recruitment of study participants as one of the primary goals of studies was indirectly expressed as one of their major achievements in CE.

“We have never failed to get a [needed] sample [size] and scientists have never failed to get samples they need from our participants because of that [community engagement].” CLO-02.

For all biomedical studies involving human participants, being able to retain the enrolled study participants is critical for the study’s success. In the view of one of the PIs, for that matter, CE is valuable as a strategy for identifying and addressing obstacles to the retention of study participants:

“[…] it is one thing screening and enrolling people but are you going to retain them in the study because as you enroll them you get to realize there are different challenges that may affect their retention so you need […] to work closely with the community engagement […].”(PI-01).

### Means of conducting CE

With regard to the most effective strategies, methods or approaches that could be used in undertaking CE, it emerged that ‘no size fits all’. Which approach to use will vary with geographical contexts even within the same country (including in the same community), and with the nature of studies including the goals and objectives of the study, target populations, among others. Even though there is noteworthy overlap between the approaches and methods used by different research projects, our respondents indicated that it would be difficult and, in any case undesirable, to assign privilege to any of them over the others. For this reason, our respondents suggested first and foremost that it may be more rewarding to propose criteria to use whenever trying to identify the most appropriate approaches, strategies, activities and methods, as opposed to recommending and ranking specific methods, approaches, strategies and activities. The following were the dominant views on the factors to consider in choosing which methods/approaches/strategies are appropriate for CE in different contexts and studies.

Resources Implications: A majority of our respondents emphasized the caution that each approach, method or strategy, along with its activities or mechanisms chosen to implement CE, has varying resource implications. Hence, in our respondents’ view, the success and failure of any CE may depend on whether there are sufficient resources to implement the chosen activities and methods used to implement them. This view is represented by the following quote:

“So you must consider the budget also for example if you say let us put announcement may be in New Vision or Bukedde [some of Uganda’s major national Newspapers], what is the price, is the project okay with it?” FG03-08.

On the other hand, activities such as community mobilization and education, literacy levels and things such as the reading culture of the target community especially of the potential study participants, were widely indicated as critical factors to consider in choosing communication mechanisms during CE:

“But also you know reading Newspapers is a problem because they use it to light ‘sigiri’ [Trans: Charcoal stove], or toilet paper instead of reading so that may not work for village community because of illiterate participants.” FG03-08.

Settlement patterns and population densities in target communities were also described as critical consideration in choosing the means:

“[…] for example in islands it is like a town. People are near [each other] so a community radio [Megaphone] can reach all but you cannot use the same in the village like you [the interviewer] have said you come from Mbarara we hear people in the whole parish you have maybe 20 homes and because of distance you cannot use it.” FG03-07.

Some of our respondents noted that participation in some studies may lead to stigma for participants. Hence, bearing in mind the nature of different studies, mobilization, sensitization and other CE activities should take into consideration the risk of stigma to those who will eventually participate in the study. To most of our participants, being enrolled in certain studies such as HIV-related studies must be kept a secret, otherwise very few, if any, will be willing to be study participants. Their reasoning was that any CE activities or methods that may lead communities to identify or suspect anybody as a study participant would be counterproductive. For example, we heard that:

“Ya, as someone talked about stigma and rumors, if say maybe people who are like this come for a meeting on the sub-county office on Tuesday. I can assure you, you are wasting your time because it is a secret.” FG03-07.

Others indicated that considering the convenience of the community and study participants is critical. Some of the respondents indicated that agricultural seasons—such as ploughing, planting, weeding and harvesting—make a difference for especially studies that are targeting rural communities. Another factor cited in determining communities’ and participants’ convenience was time of the day and place of meeting given that a particular community is of interest:

“At times depending on the participants you want for a particular trial you may find that it is necessary to maybe just talk to bar owners so the team usually organizes a separate meeting for bar owner or you feel you just want to talk to the men, may be you’re going to find them when they are doing their [fishing] nets on their boats along the shores. […]*”* (PI-01).

Having heard the above views, we probed further to see if our respondents could help identify specific strategies, approaches and methods different CABs and research projects used in their CEs, and they believed could work best generally. However, several respondents insisted that they figure out their strategies, methods and approaches based on local circumstances. One of the views emphasized and that was never contradicted by a single respondent was that they do not usually have a list of fixed methods, strategies or approaches to use. However, they were able to state their most commonly used means of conducting CE, although with strong implicit caution that the mere fact of their common use does not suggest that they should generally be ranked highly in the choice of appropriate means to conduct CE in future studies. Further, several respondents talked about the importance of implementing several strategies or targeting strategies to the group one hopes to engage. On this issue, the most revealing response we obtained after insistently probing on the best methods, approaches and strategies was the following:

“Maybe I may not answer your question very well as you want because for us in our work we don’t have a list of approaches and, […] But I can tell you about our activities. Every day we are doing our work because even if someone comes to your shop and ask by the way I heard like this or like that; is it true? Of course, you can share there and then even in a taxi, but also we reorganized in our work because sometimes they can say, hey, we have some money for mobilization and we plan together maybe like sports like competition and the winner gets a cow. So, […] Creativity is very important.” FG03-02.

One of the PIs corroborated the same perspective in the following words:

“[…] there is no particular strategy that we use so you find that for instance you want to just go and educate communities, you will call for a meeting then you will need to have some materials, many times because these meetings don’t take place in a hall where you’re going to make a power point presentation, we usually go with the materials like the IEC materials that the sponsor sends: flip charts that are pictorial, that someone can look at and understand what you are talking about […] before we would just use the radio announcements ,we engage those local radios to go around. […]*.* (PI–03)

Although no preference was explicitly indicated for any single strategy for engaging communities, there was a view that generally face-to-face engagement is better than technology-mediated engagement; for example, community events are better than radio talk shows. According to this view, whereas in principle radios would be good for massive outreach, such technology-mediated channels are less effective compared to community-based face-to-face engagements.

Even though no specific methods, strategies, approaches and actives were indicated as generally the best, our respondents indicated some of the methods, activities, strategies or approaches they have used in their CE activities. One of such activities is periodic meetings. One of the FGD participants had this to say:

“Maybe I can just list the rest: radio announcements, megaphones maybe around the place, mass sms, some islands have community radios like megaphones but stationed in one place, ya, there many methods we use depending on many factors. Well, I can say for example if you, for example, I can say that for example if a study has stigma chances you cannot announce in church in that case you can use SMS or call them on phone, ya.” FG03-01.

Further, one of our participants from CABs indicated one innovative and clear-cut strategy for ensuring that issues are timely identified and addressed, especially those of an emergency nature. This was the formation of certain responsibility committees within communities entrusted with such responsibility:

“We have an issue management committee, an issue management committee comes in when there is an emergency, they really put in a quick intervention to see how it should be handled and thereafter it reallocates the proper solution […].” FG02-01.

Another strategy specifically for information sharing and mobilization that was widely indicated is taking advantage of religious and other social events and gatherings.

“Yes, sports are important in mobilization but let me talk about another thing, like in those days [early days of HIV/AIDS in Uganda] even in church everybody was talking about ‘silimu, silimu silimu’ [local term for HIV/AIDS] so every members got awareness so churches can help in mobilization. […] FG03-03.

“[…] community gathering even funerals, weddings, and introduction [ceremonies for giving away the bride] are good opportunities especially if you are careful.” FG03-07

Another typical strategy that was mentioned was in the form of whom to involve in CE activities. In regard to this as a strategy for ensuring successful CE, they suggested a variety of group representation—politicians, religious leaders, journalists, bar owners, etc., depending on the target population.

Generally, regarding strategies, methods, and approaches for effective community engagement, our respondents’ views implied that there is no substitute for researchers’ deeper understanding of their target communities and the local dynamics therein.

### Evaluation of CE

Given the critical importance of meaningfully engaging communities in research, it is important to deliberately and systematically track and evaluate community engagement processes. Respondents in this study suggested that some research projects have formally evaluated CE while others have not. In one project, the trigger for the evaluation was indicated to be a failure to achieve some of its key goals, specifically reaching their recruitment and retention targets. Further, even though it is expected that if there were to be any formal evaluation of CE, the project’s or institution’s CLOs would be actively involved, one of the liaisons officers indicated that these evaluations are a responsibility of the scientists.

“A formal evaluation is always done by the scientists. I was expecting to get 5000 participants in this community. Mobilization activities have been done, am failing to get the number there must be a problem in mobilization that was done, the scientists come back to us, why is this one not going on well, maybe we employ another channel. So the evaluation is done if they don’t come back to us, the evaluation is done within the quarterly meeting that’s we always hear that the target in this community was achieved.” (CLO – 02).

## Discussion

Data collection focused on three main areas which were used to code data: the goals of CE, the means and processes of CE, and the evaluation of CE. One of the important topics in CE is the question of what its goals are, or ought to be, and whether these can be generalized across settings and specific studies [1, 5, 35, 41–44]. Even though there was no agreement on a standard set of goals, we inferred from our respondents’ views four key themes which stood out, and could be regarded as universal goals of CE. These include suggestions that CE can and should contribute to the respect and promotion of communities’ agency in research; it should facilitate the achievement of the studies’ goals; improve respect, protection and promotion of community and research participants’ rights, interests and well-being; and sustain trust among the research communities. Noteworthy about the goals of CE was an overlap between the goals and the value of CE. This is similar to what is found in some of the international guidance on CE such as the Good Participatory Practices (GPP) and the Council for International Organizations of Medical Sciences (CIOMS) guideline 7 [18, 24]. According to the AVAC/UNAIDS GPP, an elaborate list of CE goals which are presented in the form of “favorable consequences” of CE include: researchers’ understanding of the health beliefs and practices of the communities; cultural norms and practices; their general familiarity with research; getting communities’ input into the design of the protocol, including the framing of an effective recruitment and informed consent process; researchers’ access to communities’ insight into the strategies for risk reduction. Other positive effects of CE include enabling researchers identify and, or developing effective methods for disseminating information about the trial and its outcomes; educating the larger communities on the proposed research; building mutual trust between researchers and the communities; agreeing on equity and eligibility criteria for participation; among others [19].

On the other hand, according to the CIOMS guideline 7, CE “is a means of ensuring the relevance of proposed research to the affected community as well as acceptance by the community”; ensuring the ethical and social value and outcome of the proposed study, and addressing issues of discrimination especially when the study involves a stigmatizing disease such as HIV [24]. However, researchers need to bear in mind that the goals identified from respondents of this study are not exhaustive of all the goals which ought to, or could be set and achieved through CE. One of such critical goals which was neither explicitly identified nor implied by our respondents is that of ensuring that studies or research questions being answered in a study are addressing the needs and priorities of the community being studied, a goal that stands out prominently in the CIOMS guideline 7 (on community engagement). Lack of an explicit mentioning of this goal can perhaps be explained by the fact that given the impact of HIV scourge in the target communities, HIV research was obviously a priority need. But also, since most of the HIV/AIDs research protocols, especially clinical trials, are developed by study sponsors, local communities have not had a chance to participate in the choice of the research questions to be answered even within HIV. This means that in trying to identify the most appropriate goals of CE for individual studies, study communities’ perspectives, scholarly views and official guidelines both local and international are complementary sources.

Noteworthy still about goals of CE, we noted that after asking our respondents what they thought the goals of CE should be, the question on the value of CE was perceived as repetitious, as they would refer us back to the responses they had given on the goals of CE. Consequently, in trying to figure out what goals of CE should and could be for each study, on top of focusing on the explicitly stated goals of CE in literature, researchers can be partly guided by what has generally been regarded as the value or importance of CE, or what has been described by the AVAC/UNAIDS GPP as the “favorable consequences” of good participatory practices.

With regards to the means of effectively conducting CE, generally, a lot of guidance has been given. Some of this guidance has come under the titles of ‘strategies’ of CE [37, 42, 45]; ‘approaches’ to CE [21, 27, 46]; while some under ‘methods’ of CE [3, 31]. However, the findings of this study indicate that what matters is not any of this nomenclature, but their essence – what do you want to do, and how do you do it in order to achieve your CE goals. Consequently, in their search for guidance on the means to plan and implement CE, researchers should treat all the views with the nomenclature just cited above as essentially the same as opposed to referring to different things. It is for this reason that for clarity, we preferred the phrase ‘the *means* of conducting CE, to represent the various nomenclatures on how best CE can be undertaken.

What is even more remarkable is that our respondents universally expressed the view that there is no need for pre-conceived approaches to or methods of conducting CE. They emphasized the need for what, in our view, are creativity, flexibility, reflexivity, and generally, sensitivity to local contexts, as well as taking into consideration the nature and aims of individual study projects or programs. So, even though researchers, especially those not familiar with CE, are highly encouraged to survey literature on methods, strategies, approaches et cetera of CE, eventually decisions on how best to conduct successful CE must take cognizance of the nature of their studies and the local nuances of the target research communities. This view was emphasized especially given the very cautious responses we received when we probed our respondents to recommend at least a few methods and approaches they considered best generally. In our opinion, a response such as “Maybe I may not answer your question very well as you want because for us in our work we don’t have a list of approaches” […] FG03-02), was a cautious affirmation of the undesirability of pre-conceived and fixed ideas about the best means to conduct CE in research.

Our findings suggested that for anyone to be able to plan and implement successful CE, there is need to be clear about what they want to achieve (goals), for example building and maintaining community trust, or ensuring sufficient recruitment and retention of study participants; what needs to be done in order to achieve that goal, for example, explaining the value and goal of the proposed study; and, later the forums, or broadly speaking, the mechanism that will be used to ensure that such information is accessed and understood by the community. This latter could, for example, be done through communicating such information at religious and other social events in the community; through mass media; channeling such information through and, or with local influencers, among others. Hence, while reviewing CE plans, reviewers may not need to insist that researchers distinguish between methods, strategies and approaches. Rather, for pragmatic reasons, focus ought to be on what the target goals of CE are, what activities will be undertaken to achieve them, how those activities will be undertaken, and finally, whether there is a clear plan for evaluating the success of the whole process of CE.

Further, generally there is a widely shared view about the need for systematic evaluation of community engagement in research and some approaches to this effect have been suggested [30, 47–49]. Even though our respondents acknowledged the importance of evaluating CE efforts as critical, some indicated that in practice this is intermittently done, and, unfortunately only when there seem to be problems with recruitment and retention of study participants. Some of the proposed approaches to CE evaluation indicate 9–13 variables which should be the focus of evaluation, covering everything that can be regarded as the goals or desirable consequences of conducting CE [30, 47]. If recruitment and retention were to be the only or dominant variables of focus in CE evaluations as suggested by some views in this study, it would be quite unfortunate given that there are several critical goals of CE. Since in our opinion the *raison d’etre* for CE in research is to balance the interests of research communities and science, it is important that researchers be more consistent and broad enough in evaluating their CE to cover all goals of CE and the means used to achieve them. Even though generally monitoring and evaluation is a complex process, at the very least we propose that researchers should: (a) Systematically and continuously reflect on, and document their results on intended and unintended positive and negative outcomes; (b) Possible reasons for these outcomes; (c) the effectiveness of the means used; (d) Community’s feedback; and (e) Lessons learned.

A potentially controversial finding of this study, however, is the ethical limits of the role of the CE teams, especially the CAB members. Both CAB members and one of the PIs indicated that CAB members get actively involved in community mobilization, sensitization, and recruitment of study participants and ensuring that such participants are retained. This raises the question of the ethical limits of the roles of CABs or of CE teams as critical structures in CE. For example, does it matter ethically if the CAB members or other CE team members were to get involved in actual recruitment of study participants or to ensure that participants remain in the study? If so, should they be classified as members of the research staff, rather than as community advisors? What could be done by CAB or CE team members to ensure retention of study participants, especially if the participants could have withdrawn from the study, which may not reek of manipulation, and or undue nudging? Would it be ethically acceptable for CAB members to advise research teams on individual potential participants’ likely adherence to study procedures as this study established? In any case, should CAB or generally CE team members be allowed to identify individual participants who have enrolled in the study, especially studies participation in which may cause some participants to be discriminated and suffer stigma?

Generally, what are the ethical limits regarding activities that may be allowed as part of community engagement? This study did not generate sufficient data to answer these questions. However, given the demands of ethical research much less clinical trials, if CE is not well managed it may lead to undesirable outcomes such as compromising the privacy and confidentiality of study participants; manipulation and coercion to enroll and remain in the study; bias in the selection of the study participants through CAB members’ advice on which individuals are likely to adhere or not, among others. However, we need to state that we are not claiming that any of these negatives has widely occurred in any of the projects covered by this study. Rather these are some of the insights we gained about some of the things that could go wrong if CE process is not further deeply studied and guided.

### Limitations

This study was conducted with those involved in, and or who directly influence CE activities in HIV/AIDS biomedical research, and most of the studies considered involved HIV vaccine trials in Uganda. Hence, experiences shared by our respondents were focused on this narrow field of research. Further, the study focused on research within one country. However, in our literature review, we aimed to be as broad as possible to determine the degree to which our findings were similar to those from other contexts. Further, this study aimed at offering guidance for successful CE in research generally. However, a lot of what is presented seems more of CABs and what they do and how they do it for research institutions and in individual research projects. This is explained by the fact, given the manner in which the practice of CE in Uganda around HIV research has evolved to date, it is almost impossible to talk about CE without talking about CABs and their activities. CABs have so far been the primary structure, especially in HIV/AIDS research, through which CE is conducted. Hence, functions of CABs are almost synonymous with CE activities. However, this does not mean that CE must be conducted through formal structures called CABs. There are potentially inexhaustible mechanisms through which researchers can plan and implement CE in research. So, it is important that in planning their CE researchers explore more mechanisms for conducting CE, especially where CABs are less feasible, unnecessary or not the best or only mechanism. Finally, even though most of literature surveyed in this study is not context-limited, a lot of primary data which have shaped views in this study is characterized by local perspectives and nuances and hence there is a limit as to the extent of generalizability of these findings outside Uganda.

## Conclusion

CE in research is still an evolving practice. For this reason, there is need for experimentation, creativity and innovation on what works best and where. Even though a lot of scholarly and official guidance exists on how to ensure effective CE in research, such guidance is usually generic, especially the international guidelines and to some extent scholarly accounts of good practices in CE. Some of the guidance that may be tailored to specific communities, countries or regions may not be directly applicable in many contexts. But even within the same community, a lot may change over time that may require reinvention of the best practices in CE in those communities. Hence, there is need for context specific guidance on how CE could best be undertaken. What the results of this study reveal is that in efforts to ensure rigorous CE, there is no substitute for creativity, reflexivity and open-mindedness. In planning, reviewing and evaluating CE plans and activities, what matters is that those involved in these activities need to focus on: (1) What goals need to be achieved in CE, (2) What activities will be undertaken to achieve those goals, (3) How those activities will be undertaken, including through whom, and finally, (4) Whether there is a deliberate and elaborate plan to track the appropriateness and effectiveness of the first three. Since it is neither feasible nor necessary to have fixed ideas on what the details of each of these four items should be, how to judge the satisfactoriness of each of these, will depend on the views of investigators, community members, REC members and others involved in research planning and regulation (Additional file 1).

## Supplementary Information

**Additional file 1: Data collecting tools for the study:** Guidance for Planning, Reviewing and Assessing Community Engagement in Biomedical Research Involving Human Participants: A Case Study of the HIV/AIDS Research at UVRI in Uganda.

## Data Availability

The datasets generated and/or analyzed during the current study are not yet publicly available due to limitations of ethical approval involving the participants’ data and anonymity but are available from the corresponding author on reasonable request

## References

[1] Holzer JK, Ellis L, Merritt MW (2014). Why we need community engagement in medical research. J Investig Med.

[2] Musesengwa R, Chimbari MJ, Mukaratirwa S (2017). Initiating community engagement in an ecohealth research project in Southern Africa. Infect Dis Poverty.

[3] Pellicano E, Dinsmore A, Charman T (2014). Views on researcher-community engagement in autism research in the United Kingdom: a mixed-methods study. PLoS One.

[4] Lwin KM, Cheah PY, Cheah PK, White NJ, Day NP, Nosten F, Parker M (2014). Motivations and perceptions of community advisory boards in the ethics of medical research: the case of the Thai-Myanmar border. BMC Med Ethics.

[5] Hood NE, Brewer T, Jackson R, Wewers ME (2010). Survey of community engagement in NIH-funded research. Clin Trans Sci.

[6] Reynolds L, Sariola S. The ethics and politics of community engagement in global health research. In: Taylor & Francis. 2018.

[7] Brenner BL, Manice MP (2011). Community engagement in children's environmental health research. Mount Sinai J Med J Trans Personal Med.

[8] Pratt B, de Vries J (2018). Community engagement in global health research that advances health equity. Bioethics.

[9] Adhikari B, Pell C, Cheah PY (2020). Community engagement and ethical global health research. Global Bioethics.

[10] King KF, Kolopack P, Merritt MW, Lavery JV (2014). Community engagement and the human infrastructure of global health research. BMC medical ethics.

[11] Seeley JA, Kengeya-Kayondo JF, Mulder DW (1992). Community-based HIV/AIDS research—Whither community participation? unsolved problems in a research programme in rural Uganda. Soc Sci Med.

[12] Nakibinge S, Maher D, Katende J, Kamali A, Grosskurth H, Seeley J (2009). Community engagement in health research: two decades of experience from a research project on HIV in rural Uganda. Trop Med Int Health.

[13] Kakuhikire B, Satinsky EN, Baguma C, Rasmussen JD, Perkins JM, Gumisiriza P, Juliet M, Ayebare P, Mushavi RC, Burns BF (2021). Correlates of attendance at community engagement meetings held in advance of bio-behavioral research studies: a longitudinal, sociocentric social network study in rural Uganda. PLoS medicine.

[14] Cresswell FV, Kasibante J, Martyn EM, Tugume L, Stead G, Ssembambulidde K, Rutakingirwa MK, Kagimu E, Nsangi L, Namuju C (2020). A Journey of Hope: giving research participants a voice to share their experiences and improve community engagement around advanced HIV disease in Uganda. AAS open research.

[15] Mburu G, Iorpenda K, Muwanga F (2012). Expanding the role of community mobilization to accelerate progress towards ending vertical transmission of HIV in Uganda: the Networks model. J Int AIDS Soc.

[16] Tumwesige W, Namatovu P, Bahar OS, Byansi W, McKay MM, Ssewamala FM (2021). Engaging community and governmental partners in improving health and mental health outcomes for children and adolescents impacted by HIV/AIDS in Uganda. Pediatr Med.

[17] Uganda: National Guidelines for Research involving Humans as Research Participants. In*.* Edited by Technology UNCoSa; 2014.

[18] UNAIDS, AVAC: Good participatory practice guidelines for biomedical HIV prevention trials. 2011. In: Geneva, Switzerland, Second Google Scholar. 2014.

[19] UNAIDS, WHO: Ethical considerations in biomedical HIV prevention trials. In: Geneva: Joint United Nations Programme on HIV/AIDS (UNAIDS). 2012.

[20] Slevin KW, Ukpong M, Heise L. Community engagement in HIV prevention trials: evolution of the field and opportunities for growth. Microbicides Dev Programme aids2031 Science and Technology Working Papers. 2008.

[21] Ellen JM, Wallace M, Sawe FK, Fisher K (2010). Community engagement and investment in biomedical HIV prevention research for youth: rationale, challenges, and approaches. JAIDS J Acquir Immune Defic Syndr.

[22] Day S, Mathews A, Blumberg M, Vu T, Rennie S, Tucker JD (2020). Broadening community engagement in clinical research: designing and assessing a pilot crowdsourcing project to obtain community feedback on an HIV clinical trial. Clin Trials.

[23] van Delden JJ, van der Graaf R (2017). Revised CIOMS international ethical guidelines for health-related research involving humans. Jama.

[24] WHO, CIOMS: International ethical guidelines for health-related research involving humans: Geneva: Council for International Organizations of Medical Sciences. 2016. 40523065

[25] Tindana P, de Vries J, Campbell M, Littler K, Seeley J, Marshall P, Troyer J, Ogundipe M, Alibu VP, Yakubu A (2015). Community engagement strategies for genomic studies in Africa: a review of the literature. BMC Med Ethics.

[26] Lavery JV, Tinadana PO, Scott TW, Harrington LC, Ramsey JM, Ytuarte-Nuñez C, James AA (2010). Towards a framework for community engagement in global health research. Trends Parasitol.

[27] Lin CY, Loyola-Sanchez A, Boyling E, Barnabe C (2020). Community engagement approaches for Indigenous health research: recommendations based on an integrative review. BMJ Open.

[28] Gooding K, Makwinja R, Nyirenda D, Vincent R, Sambakunsi R (2018). Using theories of change to design monitoring and evaluation of community engagement in research: experiences from a research institute in Malawi. Wellcome Open Res.

[29] Marsh V, Kamuya D, Rowa Y, Gikonyo C, Molyneux S (2008). Beginning community engagement at a busy biomedical research programme: experiences from the KEMRI CGMRC-wellcome trust research programme, Kilifi Kenya. Soc Sci Med.

[30] Ahmed SM (2010). Palermo A-GS: community engagement in research: frameworks for education and peer review. Am J Public Health.

[31] Tindana P, Campbell M, Marshall P, Littler K, Vincent R, Seeley J, de Vries J, Kamuya D, Group HACEW (2017). Developing the science and methods of community engagement for genomic research and biobanking in Africa. Glob Health Epidemiol Genom.

[32] Lim R, Tripura R, Peto TJ, Sareth M, Sanann N, Davoeung C, Nguon C, Cheah PY. Drama as a community engagement strategy for malaria in rural Cambodia. Wellcome Open Res. 2017;2. 10.12688/wellcomeopenres.12594.2 PMC5645711 29062919

[33] Glandon D, Paina L, Alonge O, Peters DH, Bennett S (2017). 10 Best resources for community engagement in implementation research. Health Policy Plan.

[34] Musesengwa R, Chimbari MJ (2017). Experiences of community members and researchers on community engagement in an Ecohealth project in South Africa and Zimbabwe. BMC Med Ethics.

[35] Mfutso-Bengo J, Masiye F, Molyneux M, Ndebele P, Chilungo A (2008). Why do people refuse to take part in biomedical research studies? evidence from a resource-poor area. Malawi Med J.

[36] Fawcett SB, Paine-Andrews A, Francisco VT, Schultz JA, Richter KP, Lewis RK, Williams EL, Harris KJ, Berkley JY, Fisher JL (1995). Using empowerment theory in collaborative partnerships for community health and development. Am J Community psychol.

[37] Jones L, Wells K (2007). Strategies for academic and clinician engagement in community-participatory partnered research. Jama.

[38] Minkler M, Wallerstein N. Part one: introduction to community-based participatory research. Community-based participatory research for health San Francisco, CA: Jossey-Bass 2003;5–24.

[39] Moini M, Fackler-Lowrie N, Jones L. Community engagement: Moving from community involvement to community engagement—A paradigm shift. PHP Consulting. 2005.

[40] Newman PA (2006). Towards a science of community engagement. Lancet.

[41] Slack C, Wilkinson A, Salzwedel J, Ndebele P (2018). Strengthening stakeholder engagement through ethics review in biomedical HIV prevention trials: opportunities and complexities. J Int AIDS Soc.

[42] Tindana P, De Vries J, Campbell M, Littler K, Seeley J, Marshall P, Troyer J, Ogundipe M, Alibu VP, Yakubu A (2015). Community engagement strategies for genomic studies in Africa: a review of the literature. BMC Med Ethics.

[43] Dickert N, Sugarman J (2005). Ethical goals of community consultation in research. Am J Public Health.

[44] Isler MR, Miles MS, Banks B, Perreras L, Muhammad M, Parker D, Corbie-Smith G (2015). Across the miles: Process and impacts of collaboration with a rural community advisory board in HIV research. Prog Community Health Partnersh Res Educ Action.

[45] Shalowitz MU, Isacco A, Barquin N, Clark-Kauffman E, Delger P, Nelson D, Quinn A, Wagenaar KA (2009). Community-based participatory research: a review of the literature with strategies for community engagement. J Develop Behav Pediatr.

[46] Israel BA, Schulz AJ, Parker EA, Becker AB (1998). Review of community-based research: assessing partnership approaches to improve public health. Annual Rev Public Health.

[47] MacQueen KM, Bhan A, Frohlich J, Holzer J, Sugarman J (2015). Evaluating community engagement in global health research: the need for metrics. BMC Med Ethics.

[48] Goodman MS, Thompson VLS, Arroyo Johnson C, Gennarelli R, Drake BF, Bajwa P, Witherspoon M, Bowen D (2017). Evaluating community engagement in research: quantitative measure development. J Community Psychol.

[49] Haslett T, Ballenden C, Bassett L, Godbole S, Walker K. Framework for development and evaluation of community engagement. IJCA. 2011;31.

