# Supplementary material for: Managing community engagement in research in Uganda: insights from practices in HIV/AIDS research
Source: BMC Med Ethics. 2022 Jun 14;23:59. doi: 10.1186/s12910-022-00797-6 (PMC9199168; doi:10.1186/s12910-022-00797-6)
Supplement: Supplementary file 1 — Additional file 1: Data collecting tools for the study: Guidance for Planning, Reviewing and Assessing Community Engagement in Biomedical Research Involving Human Participants: A Case Study of the HIV/AIDS Research at UVRI in Uganda. [file 12910_2022_797_MOESM1_ESM.docx]

**Study Title: Planning, Reviewing and Evaluating Community Engagement in Biomedical Research Involving Human Participants**

**Matrix for Planning, Reviewing and Evaluating CE in biomedical research involving human participants**

**CE Tool 1: Officials and Regulators**

**Note: The contents of this particular tool have been deleted with the permission of the editor, given that the matrix contained in this tool is being revised as a separate publication.**

**Thanks for your participation**

**CE Tool 2: PIs and/or CE officers for the selected Projects**

**Study Title: Guidance for Planning, Reviewing and Assessing Community Engagement in Biomedical Research Involving Human Participants: A Case Study of the HIV/AIDS Research at UVRI in Uganda**

**Tool Title: Researchers’ hands-on Experience with CE in Biomedical Research**

**Discussion questions**

**Preliminaries**

1. Tell me about the history of your cab and its present role(s) in health-related research.
2. What is your general experience (length and description) with research in which CE was undertaken?
3. To what degree do you think CE is important for health-related research, given your experience as a researcher? Do you think it’s important for some studies? For all studies? Do you think it’s not actually so important? What are your thoughts?

**Goals of Community Engagement**

1. With regard to study X (selected study at UVRI), what was the goal of doing CE in this study?
2. What role did you play in the design and implementation of CE?
3. Why did you do CE? How did you expect it to affect your study?
4. What went well? What didn’t go as you planned?
5. How did doing CE change how the study ultimately was implemented?
6. Given what transpired in the study, if you were to repeat the same study, is there anything you think you may do differently? Explain.
7. Are there any goals of CE you would recommend for every study that engages in CE in Uganda? If yes, which ones and why?
8. If you were going to give advice to another researcher about to do CE for their study for the first time, what advice would you give?

**Activities of Community Engagement**

1. Well, given the goals you had for CE, what activities did you put in place? Given what your CE goals were, how did you actually do your engagement?
2. Given your experience in that study and your experience since then, do you think you could have chosen more, less than or different activities from those you chose for this study? Explain.
3. At what point(s) in the study and for how long did you actively engage in CE activities in the field/community?

**Methods/Approaches/Strategies/Styles of Community Engagement**

1. Moving away from the activities themselves, it is expected that if the activities are to be effective (lead to the desired goals), they must be undertaken using certain Methods/Approaches/Strategies/Styles. For example, community education can be undertaken through TV messages, Radio messages, Community meetings, etc. The same thing applies to consultation – for example through traditional leaders, patient groups, study participants groups or a combination of these and potentially more.
2. So, in the particular case of this study, which Methods/Approaches/Strategies/Styles did you employ and why?
3. Of the various sections of society, who did you involve in your CE activities (individuals and groups of individuals) and why?
4. Looking back at how effective you were as a team in this approach to CE, if you had a chance to replicate a similar study, would you chose exactly the same Methods/Approaches/Strategies/Styles? Explain.

**Evaluating CE**

1. Were there aspects of the study, its design, its materials, or the way it was done that changed based on the CE that was done?
2. Did you conduct any formal evaluation of your CE? If yes, how did you rate your success and on what factors do you attribute that success?
3. What are some of the generalizable challenges (those which are likely to occur in most CE) that you encountered in your CE?
4. From your general experience with CE, what good practices, opportunities, and challenges would you recommend researchers should take seriously and why?

**Other Comments and Questions**

1. As we conclude our discussion, are there any comments about the subject of our discussion and/or question you would like to share or ask? If yes, please go ahead.
2. If you happen to recall any other idea which you think is compelling about the subject of our discussion, I will be glad to receive that idea by email.

**Thank you very much for sharing your very useful ideas and for your time.**

**Study Title: Guidance for Planning, Reviewing and Assessing Community Engagement in Biomedical Research Involving Human Participants: A Case Study of the HIV/AIDS Research at UVRI in Uganda**

**Tool 3: Title: CABs Experience with CE in Biomedical Research**

**Discussion Questions**

***Introductions:*** I have already introduced myself to you and now I will give everybody a chance to introduce themselves. I propose that each introduces themselves by name, role on this CAB and any other information they want to be known about them. After this, every time each person gets an opportunity to speak, they will only repeat their role on the CAB and **NOT** their name.

Before we go to specific questions, can one of us remind us what the study was about what role was played by this CAB in that study? (One participant explains).

Is there anything very important that has been forgotten? If yes, what?

In discussing the specific questions, feel free to repeat anything that has just been said in the summary we have just heard. The summary was intended only to remind ourselves of what exactly happened.

**General participation in CE (Activities and Methods/strategies/Styles/approaches**

1. How did this CAB come together?
2. At what point of the study did you actively get involved, in what activities and for how long?
3. Why did you decide to become part of the CAB?
4. What do you see as the goal of the CAB? Why does it exist? What is its purpose for research studies?
5. What do you think the CAB does well? How well does it meet its goals? What end up being the challenges?
6. In your opinion, do you think this process could have been planned and executed in a better way than it was? Explain.
7. How do you normally ensure that your community’s interests were taken care of in the studies? Do you usually speak on behalf of your broader community or the broader community speaks to researchers through you (CAB) or even sometimes the community peaks directly to the researchers? Explain.

What were the goals of you as community representatives playing the role you played in this study? What were the activities put in place to meet those goals?

1. If you were to advise researchers who want to include CE in their projects, what would you remind them to pay attention to in their planning and implementation of CE? (I mean to say something like: researchers should be careful about…, pay attention to …, Consider …, remember … etc.).

**Goals of CE**

1. Do you think it is important for researchers to undertake CE in all health-related research like the one you took part in and other related ones? Explain?
2. Do you know the reasons why you were asked to take part in this study as an interface between the research projects as the community? Explain?
3. should researchers aim at achieving through a process like the one we are talking about? Please give reasons for each goal where possible.
4. Do you think there can be some of the goals that all researchers must aim at irrespective of the type of study and community? Why do you think so?

**Challenges and opportunities**

1. Did you encounter any challenges in this process? Explain.
2. What opportunities that exist in communities do you think researchers should be capitalized on in order to successfully plan and implement a process of community engagement?

**Outcomes/benefits to the community**

1. What do you think was improved in the study as a whole and would have been worse off if you, as a CAB, and the community had not been involved?
2. Do you remember some of the suggestions/ideas/pieces of advice etc. you gave to the project team in your interaction with them and how they reacted to those ideas? Were they implemented? Explain.
3. If a researcher wanted to do CE for the first time and had never done it and came to you for advice, what advice would you give them about how to best go about doing CE?

**Other ideas**

Is there anything else that is important that should have discussed and forgotten about?

Are there any questions?

**Okay, thank you very much for your time and very useful ideas.**
